# RCSB Protein Data Bank (RCSB.org): delivery of experimentally-determined PDB structures alongside one million computed structure models of proteins from artificial intelligence/machine learning

**DOI:** 10.1093/nar/gkac1077

**Published:** 2022-11-24

**Authors:** Stephen K Burley, Charmi Bhikadiya, Chunxiao Bi, Sebastian Bittrich, Henry Chao, Li Chen, Paul A Craig, Gregg V Crichlow, Kenneth Dalenberg, Jose M Duarte, Shuchismita Dutta, Maryam Fayazi, Zukang Feng, Justin W Flatt, Sai Ganesan, Sutapa Ghosh, David S Goodsell, Rachel Kramer Green, Vladimir Guranovic, Jeremy Henry, Brian P Hudson, Igor Khokhriakov, Catherine L Lawson, Yuhe Liang, Robert Lowe, Ezra Peisach, Irina Persikova, Dennis W Piehl, Yana Rose, Andrej Sali, Joan Segura, Monica Sekharan, Chenghua Shao, Brinda Vallat, Maria Voigt, Ben Webb, John D Westbrook, Shamara Whetstone, Jasmine Y Young, Arthur Zalevsky, Christine Zardecki

**Affiliations:** Research Collaboratory for Structural Bioinformatics Protein Data Bank, Rutgers, The State University of New Jersey, Piscataway, NJ 08854, USA; Institute for Quantitative Biomedicine, Rutgers, The State University of New Jersey, Piscataway, NJ 08854, USA; Rutgers Cancer Institute of New Jersey, New Brunswick, NJ 08901, USA; Department of Chemistry and Chemical Biology, Rutgers, The State University of New Jersey, Piscataway, NJ 08854, USA; Research Collaboratory for Structural Bioinformatics Protein Data Bank, San Diego Supercomputer Center, University of California San Diego, La Jolla, CA 92093, USA; Research Collaboratory for Structural Bioinformatics Protein Data Bank, San Diego Supercomputer Center, University of California San Diego, La Jolla, CA 92093, USA; Research Collaboratory for Structural Bioinformatics Protein Data Bank, San Diego Supercomputer Center, University of California San Diego, La Jolla, CA 92093, USA; Research Collaboratory for Structural Bioinformatics Protein Data Bank, San Diego Supercomputer Center, University of California San Diego, La Jolla, CA 92093, USA; Research Collaboratory for Structural Bioinformatics Protein Data Bank, Rutgers, The State University of New Jersey, Piscataway, NJ 08854, USA; Institute for Quantitative Biomedicine, Rutgers, The State University of New Jersey, Piscataway, NJ 08854, USA; Research Collaboratory for Structural Bioinformatics Protein Data Bank, Rutgers, The State University of New Jersey, Piscataway, NJ 08854, USA; Institute for Quantitative Biomedicine, Rutgers, The State University of New Jersey, Piscataway, NJ 08854, USA; School of Chemistry and Materials Science, Rochester Institute of Technology, Rochester, NY 14623, USA; Research Collaboratory for Structural Bioinformatics Protein Data Bank, Rutgers, The State University of New Jersey, Piscataway, NJ 08854, USA; Institute for Quantitative Biomedicine, Rutgers, The State University of New Jersey, Piscataway, NJ 08854, USA; Research Collaboratory for Structural Bioinformatics Protein Data Bank, Rutgers, The State University of New Jersey, Piscataway, NJ 08854, USA; Institute for Quantitative Biomedicine, Rutgers, The State University of New Jersey, Piscataway, NJ 08854, USA; Research Collaboratory for Structural Bioinformatics Protein Data Bank, San Diego Supercomputer Center, University of California San Diego, La Jolla, CA 92093, USA; Research Collaboratory for Structural Bioinformatics Protein Data Bank, Rutgers, The State University of New Jersey, Piscataway, NJ 08854, USA; Institute for Quantitative Biomedicine, Rutgers, The State University of New Jersey, Piscataway, NJ 08854, USA; Rutgers Cancer Institute of New Jersey, New Brunswick, NJ 08901, USA; Research Collaboratory for Structural Bioinformatics Protein Data Bank, Rutgers, The State University of New Jersey, Piscataway, NJ 08854, USA; Institute for Quantitative Biomedicine, Rutgers, The State University of New Jersey, Piscataway, NJ 08854, USA; Research Collaboratory for Structural Bioinformatics Protein Data Bank, Rutgers, The State University of New Jersey, Piscataway, NJ 08854, USA; Institute for Quantitative Biomedicine, Rutgers, The State University of New Jersey, Piscataway, NJ 08854, USA; Research Collaboratory for Structural Bioinformatics Protein Data Bank, Rutgers, The State University of New Jersey, Piscataway, NJ 08854, USA; Institute for Quantitative Biomedicine, Rutgers, The State University of New Jersey, Piscataway, NJ 08854, USA; Research Collaboratory for Structural Bioinformatics Protein Data Bank, Department of Bioengineering and Therapeutic Sciences, Department of Pharmaceutical Chemistry, Quantitative Biosciences Institute, University of California San Francisco, San Francisco, CA 94158, USA; Research Collaboratory for Structural Bioinformatics Protein Data Bank, Rutgers, The State University of New Jersey, Piscataway, NJ 08854, USA; Institute for Quantitative Biomedicine, Rutgers, The State University of New Jersey, Piscataway, NJ 08854, USA; Research Collaboratory for Structural Bioinformatics Protein Data Bank, Rutgers, The State University of New Jersey, Piscataway, NJ 08854, USA; Institute for Quantitative Biomedicine, Rutgers, The State University of New Jersey, Piscataway, NJ 08854, USA; Rutgers Cancer Institute of New Jersey, New Brunswick, NJ 08901, USA; Department of Integrative Structural and Computational Biology, The Scripps Research Institute, La Jolla, CA 92037, USA; Research Collaboratory for Structural Bioinformatics Protein Data Bank, Rutgers, The State University of New Jersey, Piscataway, NJ 08854, USA; Institute for Quantitative Biomedicine, Rutgers, The State University of New Jersey, Piscataway, NJ 08854, USA; Research Collaboratory for Structural Bioinformatics Protein Data Bank, Rutgers, The State University of New Jersey, Piscataway, NJ 08854, USA; Institute for Quantitative Biomedicine, Rutgers, The State University of New Jersey, Piscataway, NJ 08854, USA; Research Collaboratory for Structural Bioinformatics Protein Data Bank, San Diego Supercomputer Center, University of California San Diego, La Jolla, CA 92093, USA; Research Collaboratory for Structural Bioinformatics Protein Data Bank, Rutgers, The State University of New Jersey, Piscataway, NJ 08854, USA; Institute for Quantitative Biomedicine, Rutgers, The State University of New Jersey, Piscataway, NJ 08854, USA; Research Collaboratory for Structural Bioinformatics Protein Data Bank, San Diego Supercomputer Center, University of California San Diego, La Jolla, CA 92093, USA; Research Collaboratory for Structural Bioinformatics Protein Data Bank, Rutgers, The State University of New Jersey, Piscataway, NJ 08854, USA; Institute for Quantitative Biomedicine, Rutgers, The State University of New Jersey, Piscataway, NJ 08854, USA; Research Collaboratory for Structural Bioinformatics Protein Data Bank, Rutgers, The State University of New Jersey, Piscataway, NJ 08854, USA; Institute for Quantitative Biomedicine, Rutgers, The State University of New Jersey, Piscataway, NJ 08854, USA; Research Collaboratory for Structural Bioinformatics Protein Data Bank, Rutgers, The State University of New Jersey, Piscataway, NJ 08854, USA; Institute for Quantitative Biomedicine, Rutgers, The State University of New Jersey, Piscataway, NJ 08854, USA; Research Collaboratory for Structural Bioinformatics Protein Data Bank, Rutgers, The State University of New Jersey, Piscataway, NJ 08854, USA; Institute for Quantitative Biomedicine, Rutgers, The State University of New Jersey, Piscataway, NJ 08854, USA; Research Collaboratory for Structural Bioinformatics Protein Data Bank, Rutgers, The State University of New Jersey, Piscataway, NJ 08854, USA; Institute for Quantitative Biomedicine, Rutgers, The State University of New Jersey, Piscataway, NJ 08854, USA; Research Collaboratory for Structural Bioinformatics Protein Data Bank, Rutgers, The State University of New Jersey, Piscataway, NJ 08854, USA; Institute for Quantitative Biomedicine, Rutgers, The State University of New Jersey, Piscataway, NJ 08854, USA; Research Collaboratory for Structural Bioinformatics Protein Data Bank, San Diego Supercomputer Center, University of California San Diego, La Jolla, CA 92093, USA; Research Collaboratory for Structural Bioinformatics Protein Data Bank, Department of Bioengineering and Therapeutic Sciences, Department of Pharmaceutical Chemistry, Quantitative Biosciences Institute, University of California San Francisco, San Francisco, CA 94158, USA; Research Collaboratory for Structural Bioinformatics Protein Data Bank, San Diego Supercomputer Center, University of California San Diego, La Jolla, CA 92093, USA; Research Collaboratory for Structural Bioinformatics Protein Data Bank, Rutgers, The State University of New Jersey, Piscataway, NJ 08854, USA; Institute for Quantitative Biomedicine, Rutgers, The State University of New Jersey, Piscataway, NJ 08854, USA; Research Collaboratory for Structural Bioinformatics Protein Data Bank, Rutgers, The State University of New Jersey, Piscataway, NJ 08854, USA; Institute for Quantitative Biomedicine, Rutgers, The State University of New Jersey, Piscataway, NJ 08854, USA; Research Collaboratory for Structural Bioinformatics Protein Data Bank, Rutgers, The State University of New Jersey, Piscataway, NJ 08854, USA; Institute for Quantitative Biomedicine, Rutgers, The State University of New Jersey, Piscataway, NJ 08854, USA; Research Collaboratory for Structural Bioinformatics Protein Data Bank, Rutgers, The State University of New Jersey, Piscataway, NJ 08854, USA; Institute for Quantitative Biomedicine, Rutgers, The State University of New Jersey, Piscataway, NJ 08854, USA; Research Collaboratory for Structural Bioinformatics Protein Data Bank, Department of Bioengineering and Therapeutic Sciences, Department of Pharmaceutical Chemistry, Quantitative Biosciences Institute, University of California San Francisco, San Francisco, CA 94158, USA; Research Collaboratory for Structural Bioinformatics Protein Data Bank, Rutgers, The State University of New Jersey, Piscataway, NJ 08854, USA; Institute for Quantitative Biomedicine, Rutgers, The State University of New Jersey, Piscataway, NJ 08854, USA; Rutgers Cancer Institute of New Jersey, New Brunswick, NJ 08901, USA; Research Collaboratory for Structural Bioinformatics Protein Data Bank, Rutgers, The State University of New Jersey, Piscataway, NJ 08854, USA; Institute for Quantitative Biomedicine, Rutgers, The State University of New Jersey, Piscataway, NJ 08854, USA; Research Collaboratory for Structural Bioinformatics Protein Data Bank, Rutgers, The State University of New Jersey, Piscataway, NJ 08854, USA; Institute for Quantitative Biomedicine, Rutgers, The State University of New Jersey, Piscataway, NJ 08854, USA; Research Collaboratory for Structural Bioinformatics Protein Data Bank, Department of Bioengineering and Therapeutic Sciences, Department of Pharmaceutical Chemistry, Quantitative Biosciences Institute, University of California San Francisco, San Francisco, CA 94158, USA; Research Collaboratory for Structural Bioinformatics Protein Data Bank, Rutgers, The State University of New Jersey, Piscataway, NJ 08854, USA; Institute for Quantitative Biomedicine, Rutgers, The State University of New Jersey, Piscataway, NJ 08854, USA

## Abstract

The Research Collaboratory for Structural Bioinformatics Protein Data Bank (RCSB PDB), founding member of the Worldwide Protein Data Bank (wwPDB), is the US data center for the open-access PDB archive. As wwPDB-designated Archive Keeper, RCSB PDB is also responsible for PDB data security. Annually, RCSB PDB serves >10 000 depositors of three-dimensional (3D) biostructures working on all permanently inhabited continents. RCSB PDB delivers data from its research-focused RCSB.org web portal to many millions of PDB data consumers based in virtually every United Nations-recognized country, territory, etc. This Database Issue contribution describes upgrades to the research-focused RCSB.org web portal that created a one-stop-shop for open access to ∼200 000 experimentally-determined PDB structures of biological macromolecules alongside >1 000 000 incorporated Computed Structure Models (CSMs) predicted using artificial intelligence/machine learning methods. RCSB.org is a ‘living data resource.’ Every PDB structure and CSM is integrated weekly with related functional annotations from external biodata resources, providing up-to-date information for the entire corpus of 3D biostructure data freely available from RCSB.org with no usage limitations. Within RCSB.org, PDB structures and the CSMs are clearly identified as to their provenance and reliability. Both are fully searchable, and can be analyzed and visualized using the full complement of RCSB.org web portal capabilities.

## Graphical Abstract

**Graphical Abstract:**
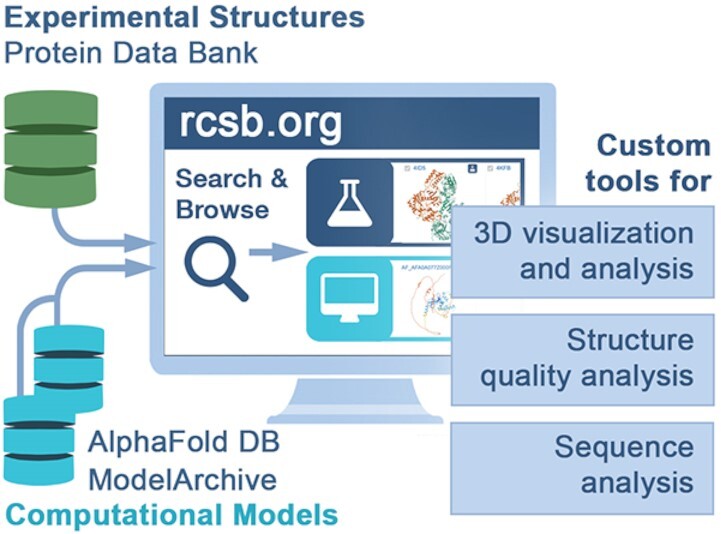
RCSB.org now delivers ∼200 000 experimentally-determined PDB structures alongside >1M Computed Structure Models that can all be searched, analyzed, visualized, and explored using custom tools and features.

## INTRODUCTION

On 20 October 2022, the Protein Data Bank (PDB) marked its 51st anniversary of continuous operations (1). As one of the most intensively used open-access biodata resources worldwide, it has been accredited by CoreTrustSeal (coretrustseal.org). In addition to the 60 000 or more structural biologists who generously contribute their data to the archive, the PDB is utilized by many millions of basic and applied researchers, educators, and students working across fundamental biology, biomedicine, bioengineering, biotechnology and energy sciences (2–28). Other database resources numbering ∼450, many of which have been highlighted in *Nucleic Acids Research* (29,30), download, integrate and distribute PDB data (30). Collectively, they enjoy open access to nearly 200 000 consistently archived, rigorously validated and expertly biocurated experimentally-determined three-dimensional (3D) structures of biological macromolecules (proteins, nucleic acids, carbohydrates) and their complexes with one another and small molecule ligands (e.g. enzyme co-factors, approved drugs, investigational agents). Because ‘function follows form’ in biology, 3D biostructures archived in the PDB have enabled myriad important scientific breakthroughs by basic and applied researchers (11,31–36).

Open access to PDB data without limitations on usage also allowed structural bioinformatics to develop as a vibrant sub-discipline of computational biology. Inspired by the work of Anfinsen who showed that the sequence of a polypeptide chain determines its shape or fold (37), members of this emerging field strove for decades to predict 3D structures of proteins accurately. Initial successes were realized using homology or comparative protein structure modeling, which depends on use of an experimentally-determined structure with a similar amino acid sequence (∼40% identity or greater) to use as a modeling template or scaffold (reviewed in (38)). As PDB archival holdings grew and the field advanced, template-free protein structure prediction became possible for very small globular proteins, fostered by two ongoing community-led blind challenges (i.e. Critical Assessment of Structure Prediction (CASP (39)), Continuous Automated Model EvaluatiOn (CAMEO (40))). The 2020 CASP challenge witnessed a sea change in structural bioinformatics. Google DeepMind emerged as the top performer with its Alpha Fold 2 software that uses artificial intelligence/machine learning (AI/ML) to predict 3D structures of proteins with accuracies comparable to that of low-resolution experimental methods (41). Subsequently, the Rosetta team led by David A. Baker (University of Washington/Howard Hughes Medical Institute) released RoseTTAFold (42), which also uses AI/ML methods to generate computed structure models (CSMs) of proteins with reported accuracies comparable to that of AlphaFold 2. At the time of writing, CSMs for nearly every protein sequence represented in UniProt (43) are publicly accessible from AlphaFold DB (41,44,45). Some CSMs generated by computational biologists operating independently of DeepMind (using RoseTTAFold, AlphaFold 2, etc.) are available from the open-access ModelArchive (modelarchive.org).

More than one million of these public-domain CSMs are now being delivered alongside ∼200 000 PDB structures by the Research Collaboratory for Structural Bioinformatics Protein Data Bank (RCSB PDB, RCSB.org (46–49)).

RCSB PDB was a founding member of the Worldwide Protein Data Bank (wwPDB, wwpdb.org) partnership (50,51), which has jointly managed the Protein Data Bank archive since 2003. Core RCSB PDB operations are funded by the National Science Foundation, National Institutes of Health, and US Department of Energy. RCSB PDB is headquartered at Rutgers, The State University of New Jersey, with additional performance sites at the University of California San Diego and the University of California San Francisco. Like its wwPDB partners, RCSB PDB is committed to the FAIR (Findability, Accessibility, Interoperability and Reusability (52)) and FACT (Fairness, Accuracy, Confidentiality and Transparency (53)) Principles emblematic of responsible data stewardship in the modern era. As the US data center of the wwPDB, RCSB PDB is responsible for managing deposition, validation, and biocuration of new experimentally-determined biostructures contributed by researchers working in the Americas and Oceania. Additional wwPDB Full Members include Protein Data Bank in Europe (PDBe, PDBe.org, (54)); Protein Data Bank Japan (PDBj, PDBj.org, (55)); the Electron Microscopy Data Bank (EMDB, emdb-empiar.org, (56,57)); and the Biological Magnetic Resonance Bank (BMRB, bmrb.io, (58,59)). Protein Data Bank China (PDBc) was recently admitted to the wwPDB as an Associate Member. In its role as wwPDB-designated PDB Archive Keeper, RCSB PDB is responsible for weekly updates of the archive and safeguarding both digital information and a physical archive of correspondence, etc. The replacement cost of the entire PDB archive is conservatively estimated at ∼US$20 billion, assuming an average cost of ∼US$100 000 for regenerating each experimental structure.

In order to continue serving the needs and interests of the diverse community of PDB users, an assortment of new features and tools have been developed and integrated into the RCSB PDB research-focused RCSB.org web portal, as described previously (47–49,60). A significant software development project was undertaken to overhaul the information management services underlying RCSB.org since our last *Nucleic Acids Research* Database Issue publication (47). In this comprehensive redesign, we developed a one-stop-shop for studying 3D biostructures by extending RCSB.org web portal functionality to support parallel delivery of more than one million CSMs publicly-available from AlphaFold DB (alphafold.ebi.ac.uk) and ModelArchive (modelarchive.org) together with nearly 200 000 experimentally-determined structures stored in the growing PDB archive. These CSMs reflect great advances made in the field and are not comparable to the theoretical models that were removed from the main PDB archive in 2002. While experimentally-determined PDB structures will remain the ‘gold standard’ at RCSB.org, integrated access to these models will be of great value to those studying 3D biological macromolecules. (N.B.: Criteria for inclusion of 3D biostructures in the PDB remain unchanged. They must be based on actual experimental measurements on sample specimens of the biological macromolecule(s) comprising the structure. For full details, see wwpdb.org.)

This initial release of one million CSMs reflects the number of models available at the time this software development project was initiated. It does not include the recent release at AlphaFold DB of a new set of CSMs corresponding to the whole non-redundant UniProt database (ca. 200 million entries).

The breakdown of CSMs currently integrated within RCSB.org is:

- From AlphaFold DB: Generated by DeepMind using AlphaFold 2
  - Model organism proteomes: 326 175 protein structures from 48 different model organisms
  - Global health proteomes: 238 274 protein structures from various disease-causing organisms
  - Swiss-Prot sequences (43): 542 380 protein structures, 430 961 of which are in addition to those already in the first two sets
  - MANE (Matched Annotation from NCBI and EMBL-EBI) sequences (61): 17 334 protein structures, 3844 of which are in addition to those from the above three sets
- From ModelArchive: 1106 models of core eukaryotic protein complexes produced by the Baker lab (62). Generated using a combination of RoseTTAFold and AlphaFold 2.

Expansion of the purview of RCSB.org was enabled by interoperation of the PDBx/mmCIF data standard, which underpins the PDB archive and RCSB.org services (see below), with the related ModelCIF data standard for CSMs (see below). RCSB.org now provides PDB data consumers with access to CSMs covering the entire human proteome, and those of many model organisms, selected pathogens, organisms relevant to bioenergy research (44), and protein complexes from select studies (62). Importantly, to maintain clear distinction between experimental structures and computational models, PDB structures and CSMs are identified as to their respective provenance and reliability. In addition to the RCSB.org web portal features described here, the newly integrated data are also available *via* RCSB PDB APIs: (Data, Search, and 1D-coordinates (63)), dramatically enhancing the ability of programmatic users to use CSMs in their workflows. An upcoming article will describe the new programmatic developments in more detail.

## RESULTS

### Motivation

**Figure 1.**
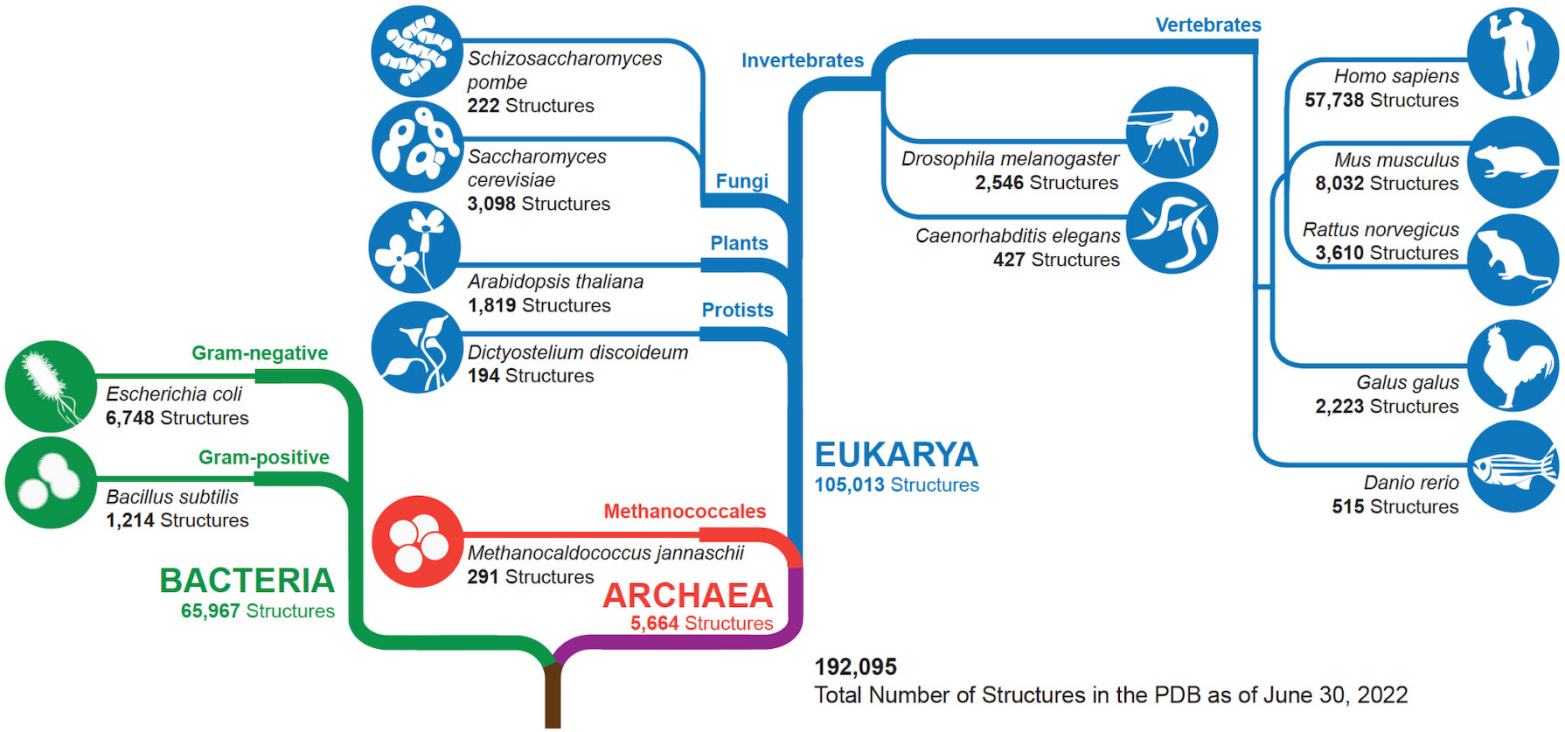
Cladogram showing PDB holdings for proteins from each kingdom of life (as of mid-2022). Within each branch, PDB structure totals are provided for selected organisms. Adapted from Figure 7 in (30). (N.B.: The PDB also houses 3D structures that solely contain nucleic acids, viral proteins, or designed proteins, which in aggregate accounted for ∼8% of archival holdings as of mid-2022.)

As of mid-2022, the PDB housed nearly 200 000 3D biostructures, encompassing proteins from organisms representing all kingdoms of life (Figure 1). Archival holdings of eukaryotic protein structures exceeded 105 000, with more than half being human in origin. Bacterial protein structures were also numerous, totaling nearly 66 000 (∼10% of which came from *E. coli*). Archaeal protein structures were the least numerous (totaling ∼5500). Notwithstanding the importance of model organisms in basic and applied research in biology, PDB coverage is decidedly limited, with mouse protein structures being most numerous at ∼8000 structures.

Rigorously validated and expertly biocurated PDB structures have been long-considered a ‘gold-standard’ in the biosciences and in-fact made AI/ML prediction of protein structures possible (64). Powerful tools developed by RCSB PDB for searching and analysis (including sequence, structure, structure motif, sequence motif), and visualization (including 1D-3D views of annotations, Mol*) help drive research and education in the biosciences worldwide.

The value of integrating PDB structures and CSMs within RCSB.org is as follows:

1. One-stop-shop data delivery should help ∼99% of RCSB.org web portal users, who are not structural biologists and are often frustrated that their protein(s) of interest is not represented in the PDB archive as an experimentally-determined structure.
2. Inclusion of CSMs provides all users with structural information for full-length polypeptide chains. Structural biologists will be able to use this information to identify, express, and purify compact globular domains that are more likely to be crystallizable for macromolecular crystallography (MX) or sufficiently soluble to study *via* solution nuclear magnetic resonance (NMR) spectroscopy. Other users will have more information with which to develop testable hypotheses and design experiments to probe the functional importance of disordered segments of polypeptide chains.
3. One-stop-shop data delivery should help structural biologists accelerate structure determination by 3D electron microscopy (3DEM) and integrative or hybrid methods.
4. All users stand to benefit from RCSB.org capabilities supporting contextual examination of CSMs through a one-stop-shop offering parallel delivery of PDB structures and CSMs.

The decision to deliver CSMs alongside PDB structures is in no way intended to send the message that they are equivalent in accuracy. Analyses carried out by RCSB PDB and published in 2022 have shown that even confidently predicted CSMs are not as accurate as experimentally-determined structures coming from macromolecular crystallography (at 3.5 Å resolution or better (65)). PDB structures should be used preferentially whenever they are available. Moreover, most CSMs publicly available at the time of writing are those of monomeric proteins (even when they are known to exist within homo- or hetero-oligomers or complex assemblies in their physiological state). Similarly, these CSMs do not typically include information about bound ligands (e.g. enzyme co-factors, substrate analogs, inhibitors, investigational agents, approved drugs, nucleic acids). Parallel delivery of PDB structures and CSMs through the RCSB.org web portal ‘one-stop shop’ should allow all users to analyze, visualize and explore CSMs in the context of experimentally-determined structures of closely-related proteins to better understand their biological and biochemical functions.

### Data Standards: Interoperating PDBx/mmCIF and ModelCIF Data Dictionaries

The PDB data architecture is defined within the PDBx/mmCIF dictionary (66–68). mmCIF is the macromolecular extension of an earlier community data standard, known as the Crystallographic Information Framework (cif.iucr.org (69)), developed under the auspices of the International Union of Crystallography to describe small molecule X-ray diffraction studies. PDBx/mmCIF is the data standard for representing and exchanging the data required for archiving and validating structures of biological macromolecules, determined using MX, NMR and 3DEM. In 2018, to enable interoperation of PDB structure data with computational models stored in the ModelArchive (modelarchive.org) and ModBase (70), a ModelCIF dictionary extension of PDBx/mmCIF was developed, adopting common data items wherever possible. ModelCIF contains definitions specific for computational modeling such as sequence alignments, coevolution data, and model quality metrics. Like PDBx/mmCIF, ModelCIF is both human- and machine-readable and fully extensible. Frameworks describing the PDBx/mmCIF and ModelCIF dictionaries are regulated by Dictionary Definition Language 2 (DDL2), a generic language that supports construction of dictionaries composed of data items grouped into categories (71). DDL2 supports primary data types (e.g. integers, real numbers and text), boundary conditions, controlled vocabularies, and linking of data items together to express relationships (e.g. parent–child related data items). DDL2 is described by its own dictionary and is, therefore, self-validating.

The PDBx/mmCIF data standard is maintained by the wwPDB in collaboration with domain experts from the structural biology community, who make up the wwPDB PDBx/mmCIF Working Group (wwpdb.org/task/mmcif). In parallel, the ModelCIF data standard is maintained by the wwPDB in collaboration with wwPDB ModelCIF Working Group domain experts recruited from the computational biology community (wwpdb.org/task/modelcif). Content dictionaries are publicly hosted on the GitHub platform (github.com/wwpdb-dictionaries/mmcif_pdbx; github.com/ihmwg/ModelCIF). PDBx/mmCIF resources support browsing and search access to definitions in both dictionaries (mmcif.wwpdb.org). A standalone Python library (github.com/ihmwg/python-modelcif), initially built to support ModelCIF within ModBase (41), has been extended to enable production of ModelCIF compliant files by protein structure prediction software tools and other modeling applications. The RCSB.org infrastructure was recently revamped to consume data compliant with PDBx/mmCIF and related ModelCIF extensions. This effort enabled expansion of the RCSB.org workflows and tools to incorporate and deliver ModelCIF compliant CSMs from AlphaFold DB and ModelArchive alongside PDB experimental structures.

### Data integration: combining external information with both PDB structures and CSMs

The RCSB.org web portal provides added value to users going well beyond the content of the archive itself. In addition to serving 3D structural data, their supporting data files, and metadata, RCSB PDB integrates information from trusted data resources (Table 1) to provide insights and details about the chemistry, sequence, 3D structure determination method, structure, functions, and evolution of the molecule(s) being studied. These data, annotations, and classifications also provide contexts for applying this knowledge to address questions in biology, medicine, bioenergy, biotechnology, evolution and more. Integrating 3D structure data with external information ensures that the RCSB.org web portal operates as a ‘living data resource’. It is not uncommon for new biological or biochemical functions of a macromolecule to come to light, or new disease-causing mutations to be identified after 3D structure data are deposited to PDB or repositories for CSMs. New findings are integrated within RCSB.org on a weekly basis, thereby ensuring public access to current information. When multiple reliable data resources provide annotations/information about a specific biomolecular property, feature, or function, the RCSB PDB integrates all relevant data together with suitable provenance. Access to these data, enables users to identify and explore details that best meet their research interests/needs. For example, currently membrane protein annotations are integrated from four different data resources.

**Table 1.** Trusted external resources/data content integrated weekly with PDB archival data. This list is updated and maintained at RCSB.org (rcsb.org/docs/general-help/data-from-external-resources-integrated-into-rcsb-pdb)

Resource	Description
*Chemical Details*	
Cambridge Structural Database (72)	Crystallographic small molecule data from the Cambridge Crystallographic Data Centre
ChEBI (73)	Chemical entities of biological interest
PubChem (74)	Chemical information
*Functional Details*	
Binding MOAD (75)	Binding affinities
BindingDB (76)	Binding affinities
ExplorEnz (77)	IUBMB Enzyme nomenclature and classification
Gencode (78)	Gene structure data; Human and Mouse Gene annotations
GeneOntology (79)	Gene structure data; organization of biological data related to molecular functions, cellular components, and biological processes
Genotype-Tissue Expression (GTEx) (80)	Tissue-specific gene expression data
Human Gene Nomenclature Committee (genenames.org)	Human gene name nomenclature and genomic information
IMGT (81)	International ImMunoGeneTics information system
Immune Epitope Database (82)	Antibody and T cell epitopes
InterPro (83)	Classification of Protein Families
MemProtMD (84)	Database of Membrane Proteins Embedded in Lipid Bilayers
Mpstruc (blanco.biomol.uci.edu/mpstruc)	Classification of transmembrane protein structures
NCBI Taxonomy (85)	Organism classification
OPM (86)	Orientations of Proteins in Membranes database; Classification of transmembrane protein structures and membrane segments
PDBbind-CN (87)	Binding affinities
PDBTM (88)	Protein Data Bank of Transmembrane Proteins
Pfam (89)	Protein families
PubMed (85)	Citation information
PubMedCentral (85)	Open access literature
SAbDab (90)	The Structural Antibody Database
*Function Details (Applications)*	
ATC	Anatomical Therapeutic Chemical (ATC) Classification System from World Health Organization
ChEMBL (91)	Manually curated database of bioactive molecules with drug-like properties
DrugBank (92)	Drug and drug target data
International Mouse Phenotyping Consortium (mousephenotype.org)	Mouse gene phenotype data
Pharos (93)	Drug targets and diseases
Thera-SAbDab (94)	Therapeutic Structural Antibody Database
*Sequence Details*	
NCBIGene (85)	Gene info, reference sequences, etc.
RESID (95)	Protein modifications
SIFTS(96)	Structure Integration with Function, Taxonomy, and Sequence
UniProt (43)	Protein sequences and annotations
*Sequence Details (Glycans)*	
GlyCosmos (97)	Web portal integrating the glycosciences with the life sciences
GlyGen (98)	Data integration and dissemination resource for carbohydrates and glycoconjugates
GlyTouCan (99)	Glycan structure repository
*Structure Details*	
NDB (100)	Experimentally-determined nucleic acids and complex assemblies
PDBflex (101)	Protein structure flexibility
*Structure Details (Classification)*	
CATH(102)	Protein structure classification (Class, Architecture, Topology/fold, and Homologous superfamily)
ECOD (103)	Evolutionary Classification of Protein Domains
SCOP (104)	Structural Classification of Proteins
SCOPe (105)	Structural Classification of Proteins — extended
*Structure Determination Details*	
AlphaFold DB (41,44)	Computed Structure Models by AlphaFold 2
BMRB (59)	BMRB-to-PDB mappings
EMDB (57)	3DEM density maps and associated metadata
ModelArchive (modelarchive.org)	Computed Structure Models (e.g. by RoseTTAFold)
ProteinDiffraction.org (proteindiffraction.org)	Diffraction images
RECOORD (106)	NMR structure ensembles
SBGrid (107)	Structural Biology Data Grid diffraction images

### Searching, analyzing, visualizing, and exploring PDB structures and CSMs with RCSB.org

**Figure 2.**
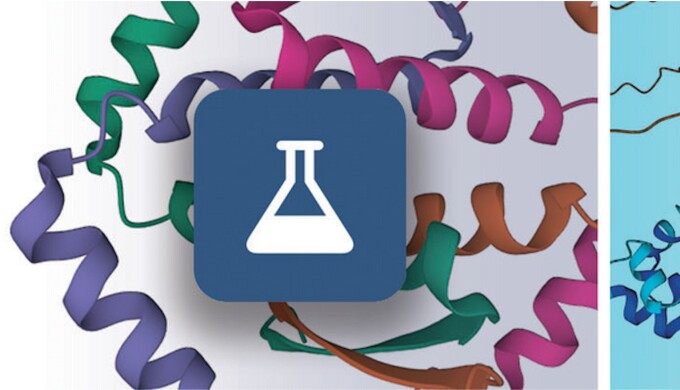
Within RCSB.org, an Erlenmeyer flask icon on a dark-blue background is used to denote experimentally-determined PDB structures (left) and a computer screen icon on a cyan background denotes CSMs (right).

Upon reaching the RCSB.org home page, users can query, organize, visualize, analyze, compare and explore PDB structures and CSMs side-by-side. Searching 3D structure information can encompass PDB structures *and* CSMs or be limited to PDB structures *only*. Either PDB structures *or* CSMs can be excluded from the search results. The two types of structure information accessible *via* RCSB.org are clearly distinguished from each other (Figure 2).

#### Top bar searching and data delivery for PDB structures and CSMs

**Figure 3.**
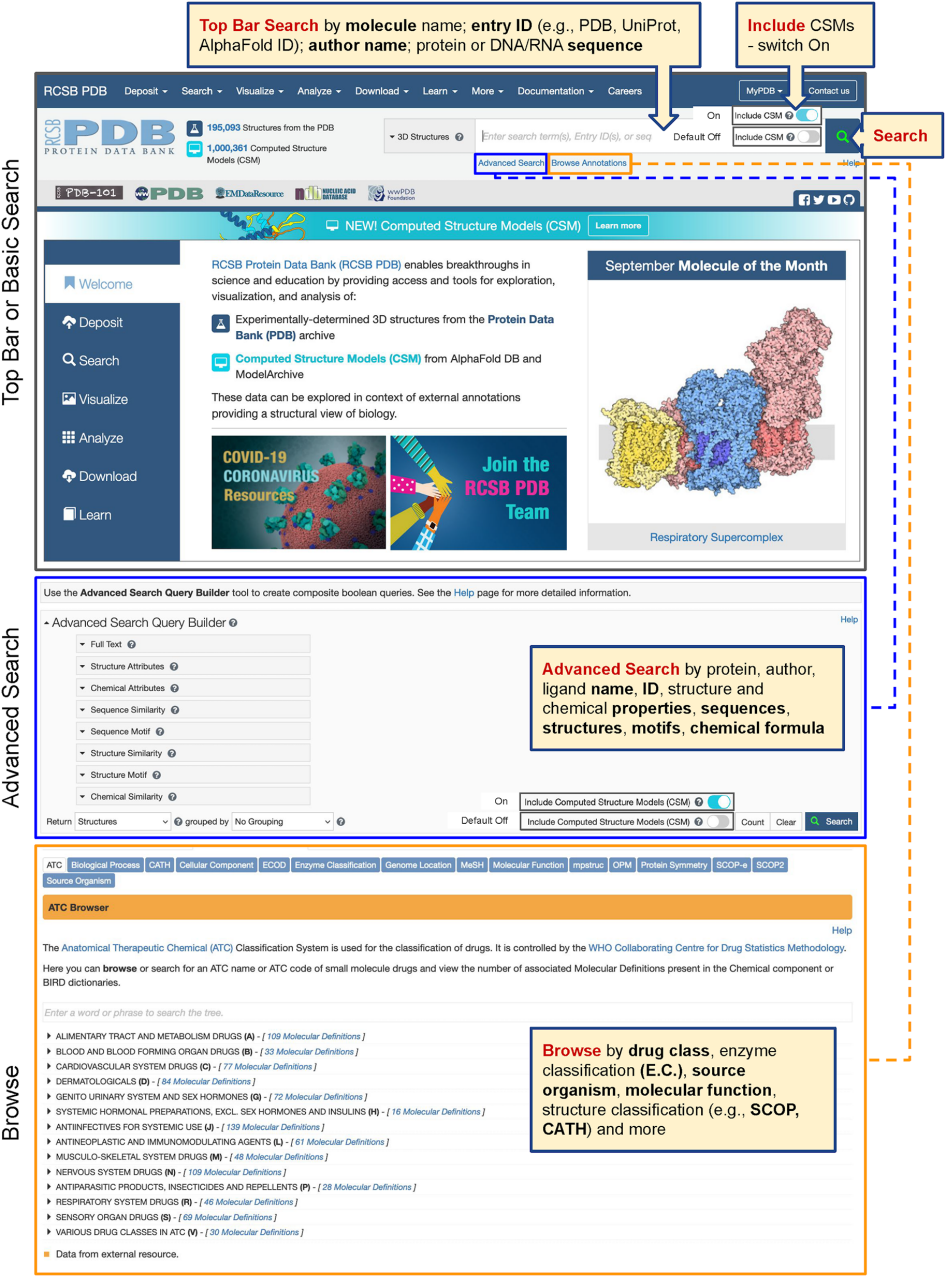
Search options at RCSB.org include Top Bar or Basic Search; Advanced Search; and Browse Annotations.

Figure 3 identifies key navigational features that provide users with access to Top Bar Search on the RCSB.org home page (top, upper panel), Advanced Search (middle panel), and Browse Annotations (lower panel).

**Figure 4.**
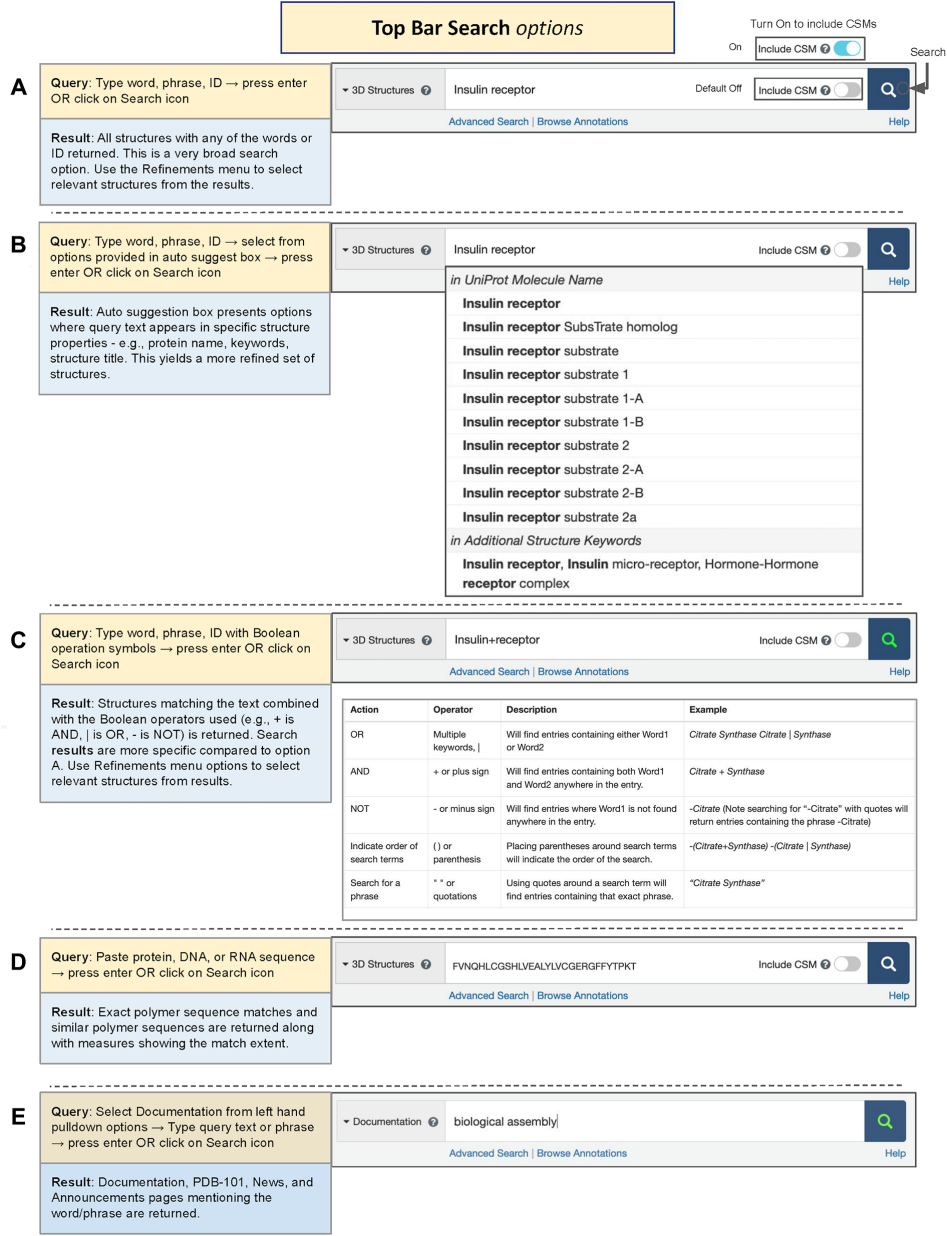
Top Bar or Basic Search options available from every RCSB.org web page. Examples of searching for 3D structures using (**A**) simple text string insulin receptor; (**B**) drop down autosuggestions based on the text string insulin receptor; (**C**) Boolean operators to combine insulin + receptor (+ = AND); or (**D**) an amino acid sequence. (**E**) Searching RCSB.org documentation using a text string biological assembly.

Top Bar Search (also referred to as Basic Search) appears throughout the RCSB.org portal (Figure 4). The default option (3D Structures) searches PDB structure data *only*; CSMs can be included when the toggle switch is activated and its color changes from gray to cyan. Entering a keyword (e.g. molecule name, database entry ID (PDB, UniProt, AlphaFold DB, ModelArchive), author name (PDB structures only)) will launch autosuggestions organized by data category. Select one of the suggestions to launch the search. Note that when using CSM identifiers (e.g. from AlphaFold DB or ModelArchive) the toggle switch to include CSMs must be activated.

Sequence searches can be run by entering single-letter code sequences for protein, DNA or RNA polymers and executing the query (hitting return or clicking the magnifying glass icon). This sequence-based search uses the MMseq2 software (108) to identify similar protein or nucleic acid sequences.

Another option supports free text searches, which are carried out most expeditiously when the phrase of interest is enclosed within double quotes. Otherwise, structures containing any of the text words in the query will be returned and may include false positives.

Top Bar Search can also be used to search documentation and news announcements available on both RCSB.org and the RCSB PDB outreach and education web portal (PDB101.RCSB.org, (109)) by changing the search type from 3D Structures to Documentation on the left of the search box.

#### Structure summary page for analyzing, visualizing, and exploring a PDB structure

To access the Structure Summary page for a PDB structure, enter the PDB structure identifier (or PDB ID for the entry) into the Top Bar search box. Each Structure Summary Page organizes information in the following categories: Overview, Literature, Macromolecules, Small Molecules, Experimental Data & Validation, etc. Tabs arrayed across the top of the page provide single-click access to 3D View (launches the Mol* molecular graphics viewer by default (110)), Annotations (structural and functional information integrated weekly from trusted external data resources), Experiment (structure provenance and related details), Sequence (1D views of each polymer sequence, with structure-related annotations), Genome (protein-to-gene-to-genome mapping for each polypeptide chain), Ligands (small-molecule validation) and Versions (entry versioning history). Since 2018, the wwPDB OneDep software system for structure deposition, validation, and biocuration (111–114) has supported versioning and replacement of atomic coordinates by the Depositor-of-Record to enable correction of errors, etc. (www.wwpdb.org/ftp/pdb-versioned-ftp-site (51)). Importantly, coordinate replacement does not trigger a change in the PDB ID, just the version number.

**Figure 5.**
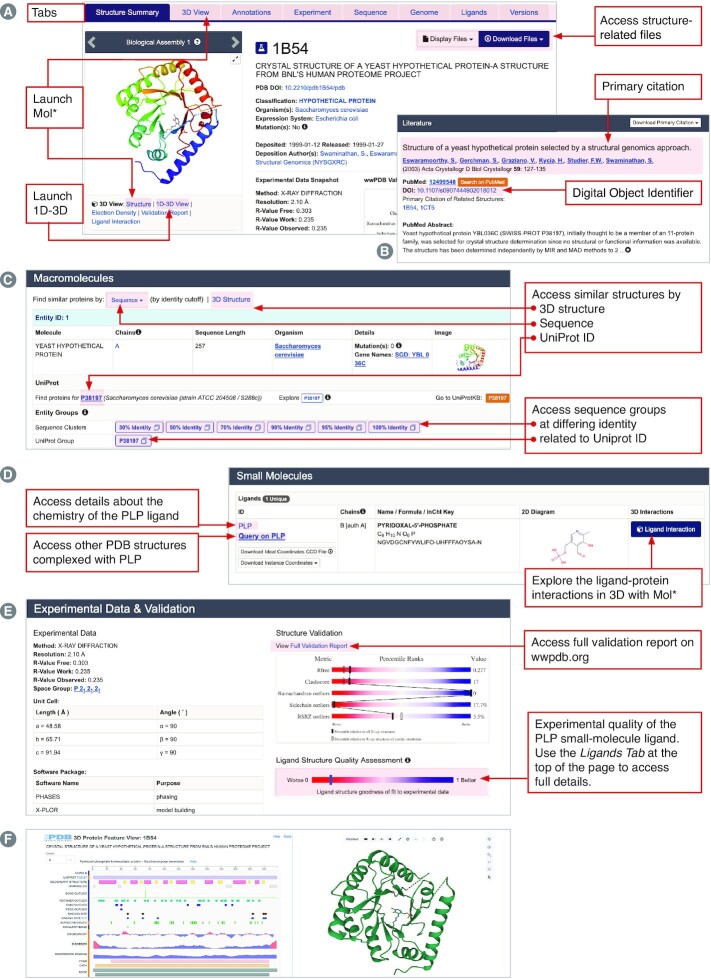
Structure Summary Page for PDB ID 1b54. (**A**) Overview. (**B**) Literature. (**C**) Macromolecules. (**D**) Small molecules. (**E**) Experimental Data & Validation. (**F**) 1D–3D Viewer.

Type the PDB ID 1b54 in the Top Bar search box to open the Structure Summary Page for the experimentally-determined structure of a ‘yeast hypothetical protein’ (Figure 5). The Structure Summary Page Overview (Figure 5A) includes entry contents, structure validation information, and access to data files. Links under the structure image will launch Mol* visualization tools (Structure, 1D-3D View, Electron Density, Validation Report and Ligand Interaction). Clicking on the 3D View tab also activates the Mol* 3D viewer for easy visualization of entire polypeptide or nucleic acid chains, whole biological assemblies (some including millions of non-hydrogen atoms), or specific atoms or groups of atoms in a particular biological macromolecule. Mol* is extensively described in RCSB.org Documentation (see below) and other publications (47,48,110,115). It operates entirely within the web browser and does not require a license, software download, or periodic update.

At the top right of the Overview section (Figure 5A), users can access drop down menus to Display Files and Download Files. For Download Files, clicking on the PDBx/mmCIF button downloads the atomic coordinates from the PDB archive in PDBx/mmCIF format (see above); atomic coordinates in Legacy PDB and PDBML/XML formats can also be accessed. Continued use of Legacy PDB format is strongly discouraged, as PDBx/mmCIF is the PDB archival format, and Legacy PDB format files may not be available for larger, more complex PDB structures.

The Literature section summarizes and provides access to the Primary Literature Citation and related information, such as the PubMed abstract (Figure 5B). When PDB structures have not been described in a scientific journal article, they may be cited using the PDB archive DOI (e.g. 10.2210/pdb1B54/pdb for PDB ID 1b54). Within the Structure Summary Page for PDB ID 1b54, the structure of the seleno-methionine form of the same protein reported in the same publication is shown (PDB ID 1cts). Clicking on the Digital Object Identifier (DOI) opens a window providing access to the journal article (in this case (116)). As of mid-2022, 162 262 PDB structures (∼84% of the entire PDB archive) had been described in 75 497 unique primary publications, the vast majority of which appeared in peer-reviewed journals. Citation analyses carried out using EuropePMC revealed that the PDB was mentioned by name in 23 030 publications in 2021, and further documented that PDB IDs were mentioned in 585 903 publications during the same calendar year.

Additional buttons provide single-click access to useful features throughout the Structure Summary Page. In the Macromolecules section, clicking on the Sequence button (Figure 5C, highlighted in pink) and selecting a sequence identity percentage returns PDB structures with similar sequences. By default, the search results returned only include PDB structures that match the query criteria. The search can be re-run to include CSMs by scrolling up to the Advanced Search Query Builder, where all the query parameters are already shown and turning on the ‘Include CSMs’ toggle switch. At the time of writing, selecting 30% sequence identity for PDB ID 1b54 returned 17 PDB structures and 67 CSMs. These search results included 3D structure information for proteins with related sequence/structure and, possibly, biochemical function from many organisms ranging from human to *E. coli*. When the 30% sequence identity search was limited to PDB structures, only data for *S. cerevisiae* and *E. coli* were returned. More distantly-related PDB structures and CSMs can be identified using the 3D Structure search button (Figure 5C), which utilizes a computationally-efficient method based on Zernike polynomials to provide real-time 3D structure similarity searches across the entire PDB archive (117) and now across all integrated CSMs (when the ‘Include CSMs’ toggle switch is turned on). At the time of writing, 82 CSMs with a similar structure to PDB ID 1b54 were found. Single-click search buttons are also available in the Macromolecules section to enable searching for PDB structures that include any part of the amino acid sequence corresponding to the UniProt ID for macromolecules in PDB ID 1b54 (i.e. UniProt accession P38197; Figure 5C). Again, CSMs can be included in the search by turning on the ‘Include CSMs’ toggle switch in the Advanced Search Query Builder. Experimental structures and CSMs are organized into groups based on (i) clustering at differing levels of sequence identity of which PDB ID 1b54 is a member (Figure 5C) and (ii) match to UniProt ID P38197 (Figure 5C). Key features of these pre-computed groups may be explored on the corresponding Group Summary pages, accessible by clicking on the blue outlined boxes shown in Figure 5C. These groups can provide valuable insights about sequence conservation, ligand binding, domain/functional annotations, *etc*. The RCSB.org structure grouping feature was introduced and described in detail in (118).

The system supporting all structure searches within RCSB.org is powered by the BioZernike method (117), which can identify global structure similarities for any size structure, including any macromolecular assembly. It does so by using Zernike polynomials, which provide a means to decompose 3D volumes into descriptor vectors that can be compared very rapidly. The main drawback of this method *versus* traditional methods such as DALI is that it is not able to find local matches. Thus, it cannot find two proteins that share a similar domain structure (but are otherwise globally different). However, it offers two very important advantages: (a) it can search across large numbers of structures, requiring less than a second for the entire PDB archive *versus* minutes to hours for DALI and (b) it also works for assemblies.

The Small Molecules section of the Structure Summary Page is shown in Figure 5D. In PDB ID 1b54, a well-known enzyme co-factor (pyridoxal phosphate, wwPDB Chemical Component Dictionary ID PLP) was copurified with the yeast hypothetical protein expressed in *E. coli* and revealed during experimental structure determination. Full details regarding the chemistry of PLP can be accessed by clicking on PLP (Figure 5D). Clicking on Query on PLP (Figure 5D) invokes a search that returns all PDB structures that contain PLP as a bound small-molecule ligand (1212 at the time of writing). The environment of PLP in PDB entry 1b54 can be viewed using Mol* by clicking the Ligand Interaction button (Figure 5D), which reveals a covalent bond between PLP and the sidechain of lysine 49 (see below).

The Experimental Data & Validation section (Figure 5E) provides a summary of macromolecular structure determination results, summary sliders that indicate experimental structure ligand quality, and access to the wwPDB Validation Report (Figure 5E). Details regarding the quality of the PLP small-molecule ligand detected in the experimental electron density map can be found in the wwPDB validation report and accessed graphically by clicking the Ligands tab at the top of the Structure Summary Page (65).

#### Structure Summary Page for analyzing, visualizing and exploring a CSM

Top Bar or Basic search can be used to find CSMs once the ‘Include Computed Structure Models (CSM)’ switch is turned on (i.e. it appears in cyan not gray). Supported searches include single-letter code amino acid sequence and by ID (AlphaFold DB, ModelArchive, and UniProt). CSMs have two IDs that are searchable on RCSB.org: the original modifier assigned by the source database and slightly modified IDs employed within RCSB.org that uses a prefix (AF_ or MA_, indicating the source database); the ID is displayed in capital letters, and hyphens have been removed (e.g. AlphaFold DB identifier AF-B3EWR1-F1 is represented as AF_AFB3EWR1F1).

**Figure 6.**
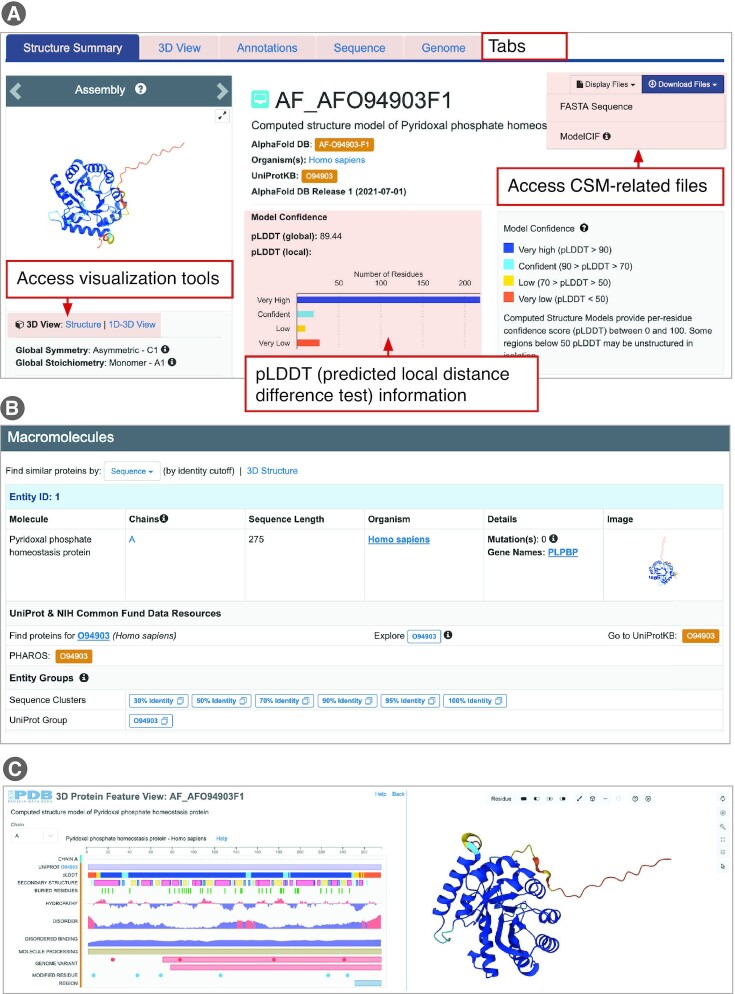
Structure Summary Page for the AlphaFold DB CSM AF_AFO94903F1. (**A**) Overview (including Model Confidence). (**B**) Macromolecules. (**C**) 1D–3D View launched from the Structure Summary Page.

UniProt ID searches can be very efficient. For example, a Top Bar search (including CSMs) for UniProt ID O94903, a pyridoxal phosphate homeostasis protein and human homolog of PDB entry 1b54, returns the appropriately matched CSM entry from AlphaFold DB (at the time of writing). Note that the results list and corresponding Structure Summary Page (Figure 6) displays the RCSB.org assigned identifier (AF_AFO94903F1). This RCSB.org-assigned ID is also used in various search, visualization, and structure comparison tools available from RCSB.org. The AlphaFold DB ID for this CSM (AF-O94903-F1) is included on the orange-colored box linking users to the source database. Albeit somewhat simplified, CSM Structure Summary Pages have the same look and feel as those describing PDB structures. Two sections are currently included CSM structure summary pages, including Overview and Macromolecules. Tabs arrayed across the top of the page provide single button click access to 3D View (Mol* molecular graphics viewer), Sequence (1D views of each polymer sequence, with structure-related annotations), and Genome (protein-to-gene-to-genome mapping for each polypeptide chain). At the top right of the Overview section (Figure 6A), users can access the Display Files and Download Files drop down menus. For Download Files, clicking on the ModelCIF button downloads the atomic coordinates from either AlphaFold DB or ModelArchive in ModelCIF format (see above). CSM provenance information is provided within the Overview section. The Macromolecules section (Figure 6B) supports all the functionality represented in the Macromolecules section of the Structure Summary Page of an experimentally-determined PDB structure.

Of particular importance when evaluating CSMs for use in research and education are pLDDT (predicted local distance difference test) scores or confidence estimates generated by AlphaFold 2 (41,119,120). pLDDT scores (values between 0 and 100) denote polypeptide chain segments as very high confidence (pLDDT$ \ge$90), confident (90 > pLDDT$ \ge$70), low confidence (70 > pLDDT$ \ge$50), and very low confidence (pLDDT < 50). Within CSM Structure Summary Pages, model confidence information is provided in the Overview section (Figure 6A). Both the global pLDDT score (∼89 for this example, indicating a Very High Confidence prediction) for the CSM and a histogram of per amino acid residue local pLDDT scores are provided. For CSM ID AF_AFO94903F1, the histogram shows that 238 of the 275 residues have either Confident or Very High Confidence pLDDT scores. Visual inspection of the CSM with the Mol* graphical display feature, accessible by clicking Structure in the 3D View box on the left of Figure 6A, reveals that these Low (color coded yellow) and Very Low Confidence (orange) segments of the polypeptide chain correspond to the N- and C-termini of the CSM. In contrast, residues 12–249, corresponding to the globular portion of the CSM, have either Confident (cyan) or Very High Confidence (blue) pLDDT scores. In addition, the RCSB.org 1D–3D View provides an integrative view of the local scores with other biological annotations at sequence and structure levels (Figure 6C). For CSM ID AF_AFO94903F1, this view shows a possibly disordered region located within the C-terminal portion of the protein with very low pLDDT scores.

#### Pairwise Structure Alignment for comparing CSMs and PDB structures

**Figure 7.**
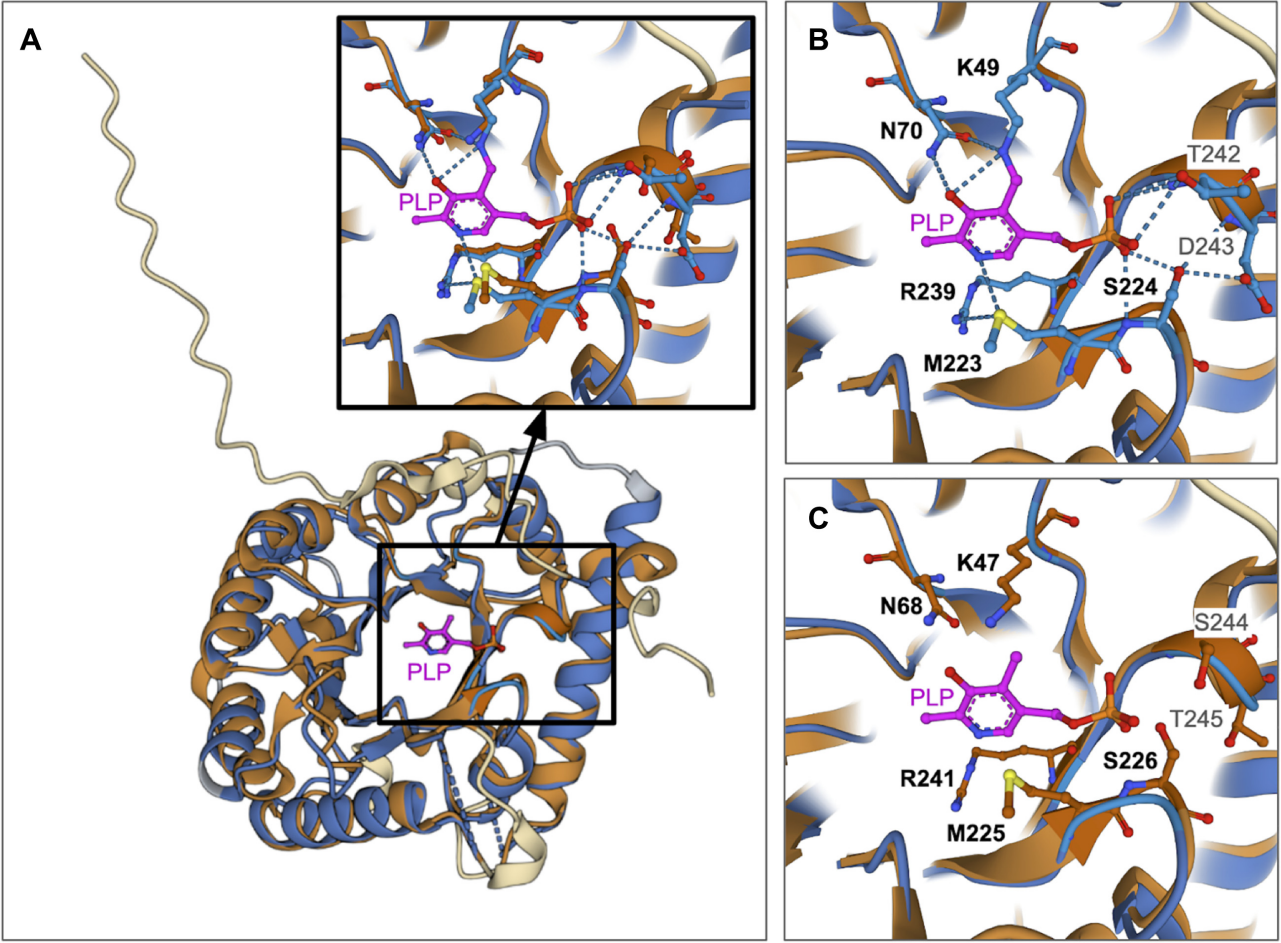
Pairwise superposition of CSM ID AF_AFO94903F1 and PDB ID 1b54. (**A**) Pair of aligned structures, with both polypeptide chains rendered using ribbon representations. Aligned portions of the PDB structure and CSM are color-coded blue and brown, respectively. Dashed blue lines represent parts of the polypeptide chain not resolved in the X-ray crystallographic experiment. Portions of the PDB structure and CSM that could not be aligned are color-coded gray and cream, respectively. PLP is shown in magenta ball-and-stick, and water molecules are shown as gray spheres. Inset is a closeup of the amino acid residues within 5 Å of the ligand in both the PDB structure and CSM. (**B**) Same view as in A-inset but showing the amino acid side chains from PDB ID 1b54 that interact with PLP. (**C**) Same view as in A-inset but showing amino acids from the CSM corresponding to the residues shown in panel (B). Conserved amino acids shown in panels (B) and (C) are identified in bold font. Atom colored coding: C-light blue, brown or magenta; N-dark blue; O-red; S-yellow. Dotted blue lines denote hydrogen bonds and charge–dipole interactions.

The Pairwise Structure Alignment tool (48), which is accessible from the Analyze drop-down menu in the RCSB.org header, can be used to compare structures (PDB experimental structures and/or CSMs) in 3D. This tool was recently augmented to support simultaneous comparison of more than two PDB structures and/or CSMs. Comparison of CSM ID AF_AFO94903F1 with the experimentally-determined PDB structure of its *S. cerevisiae* homolog shows that the structure-based sequence alignment spans residues Asp10-Gly246 of PDB ID 1b54 and Ser8 to Gly248 of the human CSM, yielding sequence identity of ∼41% with root-mean-square-deviation of ∼1.8 Å for 221 pairs of C$\alpha$ atoms. When ‘Structures’ is chosen from the Select View drop-down menu at the top of the Mol* window, a ribbon representation superposition is displayed together with water molecules (small spheres, Figure 7A) and the bound PLP ligand (ball and stick figure, Figure 7A). Clicking on the ligand allows the user to visually inspect its immediate environment with non-hydrogen atoms displayed for the CSM or the PDB structure (Figure 7B, and C, respectively). The lysine residues to which the co-factor is covalently bound in PDB structure 1b54 (*Sc*-Lys49) and the CSM *Hs*-Lys47 (*Sc* denotes *S. cerevisiae*; *Hs* denotes *H. sapiens*) occur in identical relative spatial locations. The same holds true for the following residues responsible for making non-covalent interactions with PLP in the PDB structure: *Sc*-Asn70 *versus Hs*-Asn68, *Sc*-Met223 *versus Hs*-Met225, *Sc*-Ser224 *versus Hs*-Ser226, *Sc*-Arg239 *versus Hs*-Arg241 and *Sc*-Thr242 *versus Hs*-Ser244. Taken together, these observations provide strong indirect evidence that the human homolog of the pyridoxal phosphate homeostasis protein binds PLP and does so using virtually identical covalent and non-covalent interactions to those observed in PDB ID 1b54. The position of *Hs*-Ser244 in the CSM is such that a modest rotation of the sidechain about the bond connecting C$\alpha$ and C$\beta$ would position the hydroxyl group so as to make an enthalpically-favorable sidechain dipole-to-charge interaction with the negatively-charged phosphate group of PLP (data not shown).

#### Query by Example from Structure Summary Pages

**Figure 8.**
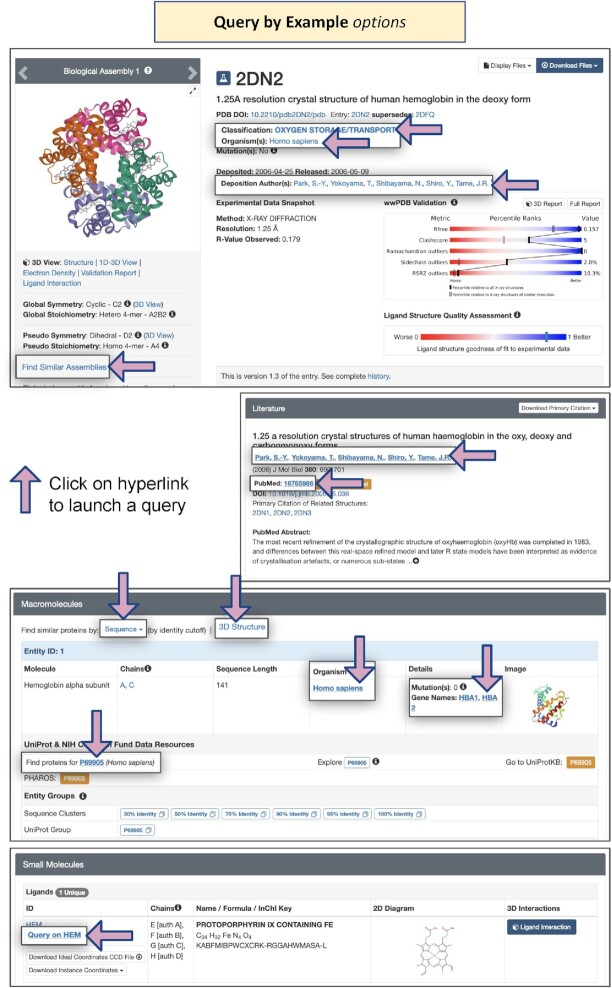
Query by Example options on Structure Summary Pages for PDB structures.

**Figure 9.**
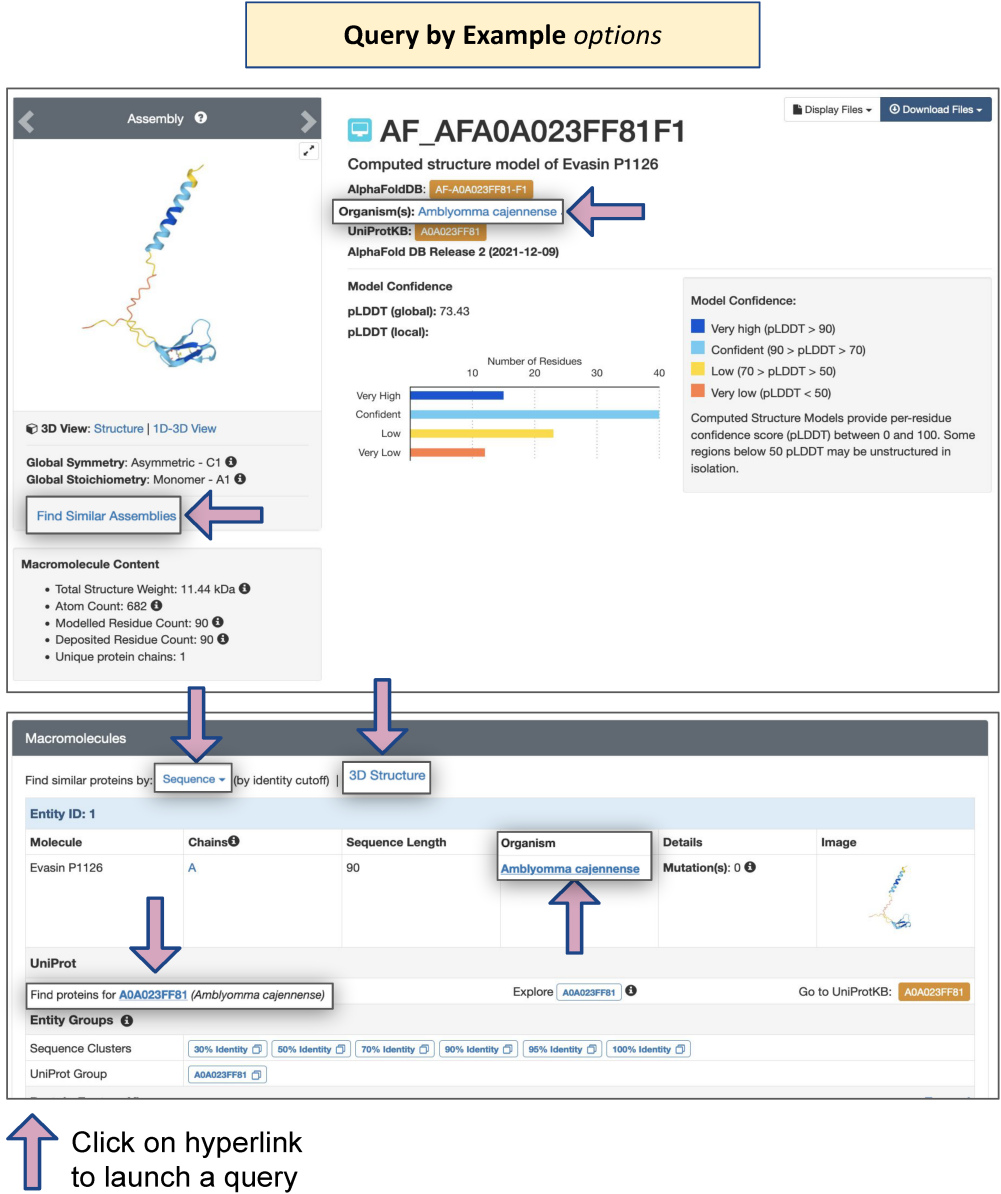
Query by Example options on Structure Summary Pages for CSMs.

Structure Summary Pages for PDB structures and CSMs support a wealth of opportunities for ‘Query by Example,’ wherein clicking on a link will launch a search for related structures. In addition to the search features described in detail above and annotated in Figures 5C–E, Figure 8 identifies locations of various useful hyperlinks on the Structure Summary Page for PDB ID 2dn2, a 1.25 Å resolution MX structure of deoxy human hemoglobin (121). Single-clicks can invoke searches for PDB structures and CSMs available from RCSB.org that are classified as being involved in Oxygen Storage/Transport, from the same Organism, with the same Deposition Author, and with similar macromolecular assemblies. (N.B.: Query by Example searches are only supported across the entire PDB archive but can be easily re-run in Advanced Search by activating the CSM toggle switch). Moving down to the Literature section, Query by Example can be used with the same journal publication (with common PubMed ID) or the same Primary Literature citation author. Within the Macromolecules section, Query by Example can also be invoked for the same Organism and the same Gene Name(s). Query by Example within the Small Molecules section for the same ligand (e.g. HEM, protoporphyrin IX containing Fe) was described above. Query by Example searches can also be launched from CSM Structure Summary Pages (Figure 9).

#### Advanced searching for PDB structures and CSMs

**Figure 10.**
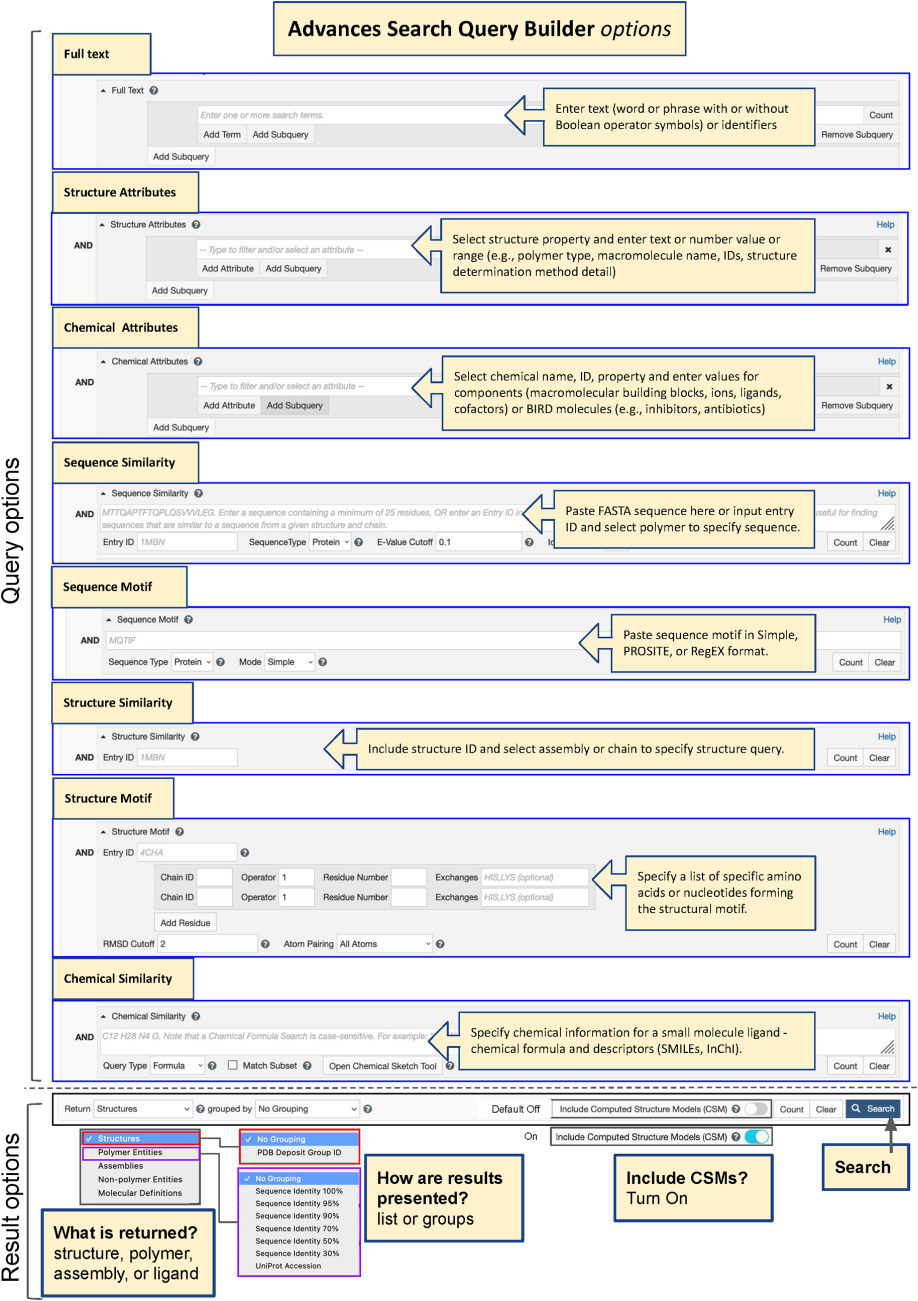
Using RCSB.org Advanced Search to construct complex Boolean queries and modify Results options.

A previous RCSB PDB article in the *Nucleic Acids Research* Database Issue described a substantial redesign of the RCSB.org web portal (47). Chief among the new website features introduced in 2021 was Advanced Searching with full Boolean logic across all data items indexed within the RCSB PDB Data Warehouse (60). Additional search capabilities reported in (48) were added after (47) was published. The purview of Advanced Search now encompasses Full Text, Structure Attributes, Chemical Attributes, Sequence Similarity, Sequence Motif, Structure Similarity, Structure Motif (122), and Chemical Similarity. With integration of CSMs into RCSB.org, Advanced Search for CSMs across Full Text, Structure Attributes, Sequence Similarity, Sequence Motif, Structure Similarity, and Structure Motif is available on an ‘opt in’ basis (as for Top Bar Search). Figure 10 provides an infographic explaining how to construct an Advanced Search Query and tailor Result options to suit.

Search attributes have been added and/or augmented in the RCSB.org Advanced Search Query Builder to support search and retrieval of CSMs, while distinguishing them clearly from PDB structures within the query results. The Structure Determination Methodology attribute is used to specifically retrieve experimental structures or CSMs. New CSM-specific Structure Attributes include Source Database, Global Quality Score—pLDDT and Computed Structure Model ID(s). Source Database enables searching for CSMs sourced from AlphaFold DB or ModelArchive. Global Quality Score—pLDDT allows identification of CSMs based on their global pLDDT score value. In addition, CSMs can be retrieved using Computed Structure Model ID(s) issued by the source database (*e.g*. AF-P00091-F1, ma-bak-cepc-0001), Entry ID(s) provided by RCSB.org (e.g. AF_AFP00091F1, MA_MABAKCEPC0001), or Accession Code(s) from other reference sequence databases such as UniProt (43) (e.g. P00091)). The Advanced Search Query Builder enables combining different attributes and different types of searches using Boolean operators (AND/OR/NOT).

### Browsing Annotations for PDB Structures

Browse Annotations (Figure 3, lower panel) enriches the RCSB.org web portal user experience by offering access to PDB structures organized by annotations under 15 categories, each accessible from its own tab. Annotations integrated from external data resources and those computed by RCSB PDB (i.e. Protein Symmetry) are identified with orange and blue banners, respectively. Under each of the tabs (Table 2), major annotation categories are listed together with the current number of related data in PDB (structures, entities, or molecular definitions). Users can select the top-level category to return search results, or drill down the different hierarchical trees for smaller data sets. A search box will autocomplete search terms with the matching classifications and highlight locations on the tree. Several of these annotations are also available from Structure Summary Pages.

**Table 2.** Browse Annotations options

Annotation	Source
ATC or Anatomical Therapeutic Chemical Classification System	WHO Collaborating Centre for Drug Statistics Methodologyhttps://www.who.int/classifications/atcddd/en/
Biological Process (vocabulary terms mapped to PDB entities by SIFTS (96,123))	Gene Ontology Consortium (79)
Class(C), Architecture(A), Topology(T) and Homologous (H) superfamilies	CATH (102)
Cellular Component locations (vocabulary terms mapped to PDB entities by SIFTS (96,123))	Gene Ontology Consortium (79)
Evolutionary Classification 0f protein Domains	ECOD (103,124)
Enzyme Classification Number	https://www.qmul.ac.uk/sbcs/iubmb/enzyme
Genome Location	UniProtKB(43), GenBank, Entrez Gene (85)
MeSH (Medical Subject Headings)	https://www.nlm.nih.gov/mesh/meshhome.html
Molecular Function	Gene Ontology Consortium (79)
Membrane Protein classification	https://blanco.biomol.uci.edu/mpstruc/
Membrane-associated protein orientation	Orientations of Proteins in Membranes (86)
Protein Symmetry calculated for all protein assemblies in PDB	RCSB PDB
Structural Classification	SCOPe (105) and SCOP2 (104,125)
Source Organism	NCBI Taxonomy (85)

### Additional Mol* 3D visualization options

The most common way to explore 3D structures available from RCSB.org is to visualize them. Within RCSB.org, 3D structures may be visualized using a web-native visualization tool known as Mol* (110,115). This tool can be accessed by clicking on the ‘Structure’ link below the thumbnail image of the structure or by clicking on the tab ‘3D View’ on the top of the page (Figure 5A). Mol* has also been implemented within other RCSB.org tools as follows:

1. Linked to a 1D (sequence) browser that can be accessed by clicking on the 1D-3D link, below the thumbnail images of the 3D structure (Figures 5A and C). This feature allows users to map and display a variety of annotations integrated from various bioinformatics resources on the 3D structure.
2. As part of the Pairwise 3D Structure Alignment tool to display regions of match between two or more structures being compared or the whole superimposed structure(s) (Figure 7). This tool can be used to compare 3D structures that are not available from the RCSB.org (e.g. 3D structure atomic coordinates stored on a local computer, using the file upload option; CSM atomic coordinates from AlphaFold DB an external data resource, using the Web Link option).

Note: In both Mol* implementations, clicking on the Expanded Viewport button in the vertical toggle menu in the Mol* 3D canvas expands the Mol* window, providing access to all options and tool functionalities.

Finally, a standalone implementation of Mol* (https://www.rcsb.org/3d-view) is available for visualizing and analyzing 3D structures not accessible within RCSB.org. The overall layout of the tool is the same with a right-hand Controls panel. The Open File options allow upload of a locally saved file, while the Download Structure options allow specification of a structural biology resource (e.g. AlphaFold DB structures not currently available from RCSB.org). Multiple structures can be uploaded to this implementation of the tool for superposition and analysis. Standalone Mol* also provides a convenient platform to upload and view a previously saved Session using the Sessions → Download/Open options.

### User documentation and introductory materials

Extensive documentation explaining use of RCSB PDB tools and resources is available from RCSB.org. Wherever possible, examples are used to illustrate the functionality of the tool/feature and relevant scenarios for which the tool may be useful. Documentation includes several General Help articles that introduce specific topics (e.g. Organization of 3D structures in the Protein Data Bank; Computed Structure Models and RCSB.org; Assessing the Quality of 3D Structures; Ligand Structure Quality in PDB Structures). In addition, articles about ‘Search and Browse’ options; ‘Exploring a Structure,’ including descriptions about the Structure Summary page; ‘3D Viewers,’ including Mol*; ‘Grouping Structure’ including descriptions of the Group Summary page; ‘Sequence Viewers,’ including Protein Feature and Genome Views; ‘Tools,’ including Pairwise Structure Alignment; plus details about programmatic access of RCSB.org data and various additional resources are available. This collection of documents is being continuously extended to reflect addition of new features and functionalities (e.g. features for CSM exploration). It is also being updated to reflect changes and improvements implemented to keep up with community needs and in response to community feedback.

For easy access, relevant RCSB.org pages provide direct links to the relevant documentation. In addition, the entire collection may be browsed (https://www.rcsb.org/docs/) or searched using the Documentation option in the Top Bar search options (Figure 4E).

## FUTURE DIRECTIONS

As the PDB archive entered its 52nd year, RCSB PDB embarked on comprehensive analyses of its diverse user communities (i.e. basic and applied researchers, educators, and students spanning fundamental biology, biomedicine, bioenergy, bioengineering and biotechnology), and strategic reviews of how it

- Delivers Data In and Data Out services efficiently to a growing user base, now numbering many millions worldwide;
- Works with wwPDB partners to process, rigorously validate, and expertly biocurate the growing number of increasingly complex PDB depositions received annually (projected at ∼16 500 for 2022);
- Manages and safeguards the growing PDB archive in its role as wwPDB-designated Archive Keeper;
- Enables efficient searching, analysis, visualization, and exploration of hundreds of thousands of experimentally-determined PDB structures integrated with more than one million CSMs through its RCSB.org research-focused web portal; and
- Supports user training, education, and outreach through its PDB101.RCSB.org introductory web portal.

Additional challenges lying ahead for RCSB PDB include, but are by no means limited to the following:

- Rapid growth in public-domain CSMs of individual polypeptide chains, already numbering >200 million at the time of writing;
- Anticipated advances in AI/ML-based prediction of structures of multi-protein complexes and those of protein-ligand complexes;
- Continued development of biomolecular structure determination methods using X-ray Free Electron Lasers, revealing the microscopic details of chemical reactions in real time;
- Growth in the number and complexity of atomic-level cryo-electron tomography structures of macromolecular machines imaged within cryogenically preserved cells and tissues;
- Integration of PDB structures and CSMs with complementary information coming from correlative light microscopy and related imaging methods across length scales ranging from atoms to small molecules to individual biomolecules to macromolecular assemblies to organelles to cells and ultimately tissues;
- Merging of the PDB-Dev (pdb-dev.wwpdb.org) prototype archiving system for integrative (or hybrid) methods structures with the PDB archive; and
- Federating other biodata resources, such as the Small-Angle Scattering Database (SASBDB, sasbdb.org) and the Proteomics Identification Database (PRIDE, ebi.ac.uk/pride), with the PDB, EMDB and BMRB core archives jointly managed by the wwPDB partnership.

Policy changes recently promulgated by the Office of Science and Technology (OSTP) in the United States (US) are also likely to affect future RCSB PDB operations. The Executive Office of President Joe Biden has called on the federal agencies with research and development expenditures to update their public access policies as soon as possible (126), and no later than 31 December 2025, to make publications and their supporting data (*e.g*. biomolecular structure information stored in the PDB archive) resulting from federally funded research publicly accessible without an embargo on their free and public release. This announcement is expected to accelerate progress towards full open sharing of data generated with federal research funding in the United States. It will add considerable weight to awareness campaigns undertaken by non-governmental organizations such as CoreTrustSeal (coretrustseal.org) and the Global Biodata Coalition (globalbiodata.org). The recent OSTP announcement also begs the question as to how heavily-used, open-access data resources, such as the PDB archive, should be sustainably funded at levels commensurate with the central roles they play in biological and biomedical research and education ecosystems worldwide (127,128).

## DATA AVAILABILITY

No new data were generated or analysed in support of this research. Resources described are available freely at RCSB.org.
